# Antioxidant activity and phytochemical analysis of fennel seeds and flaxseed

**DOI:** 10.1002/fsn3.3165

**Published:** 2023-01-24

**Authors:** Sana Noreen, Tabussam Tufail, Huma Bader Ul Ain, Anwar Ali, Rana Muhammad Aadil, Arash Nemat, Muhammad Faisal Manzoor

**Affiliations:** ^1^ University Institute of Diet and Nutritional Sciences The University of Lahore Lahore Pakistan; ^2^ Department of Epidemiology and Health Statistics, Xiangya School of Public Health Central South University Changsha China; ^3^ National Institute of Food Science and Technology University of Agriculture Faisalabad Pakistan; ^4^ Department of Microbiology Kabul University of Medical Sciences Kabul Afghanistan; ^5^ Guangdong Provincial Key Laboratory of Intelligent Food Manufacturing Foshan University Foshan China; ^6^ School of Food Science and Engineering South China University of Technology Guangzhou China

**Keywords:** amino acids, antioxidant activity, DPPH, fennel seeds, flaxseed

## Abstract

Natural herbs are now receiving more attention due to the growing demand for their antioxidant properties. This study compared flaxseed and fennel seeds for their nutritional composition, bioactive moieties, and antioxidant activity—the study comprised two different phases. According to methods, phase I analyzed flaxseed and fennel seeds for proximate composition, mineral profile, dietary fiber, and amino acid content. In phase II, seeds were extracted using three different solvents, i.e., ethanol 80%, acetone 80%, and distilled water, to probe the total phenolic and flavonoid content. Antioxidant activity was measured using DPPH and a FRAP in the final phase. Current study revealed that flaxseed had higher protein (17.33 ± 0.02%), fat content (36.76 ± 0.02%), potassium (763.66 ± 4.04 mg/100 g), iron (5.13 ± 0.03 mg/100 g), phosphorus (581.46 ± 4.07 mg/100 g), magnesium (406.60 ± 5.12 mg/100 g), and zinc (3.30 ± 0.49 mg/100 g), respectively. In fennel seed, high dietary fiber (53.2 ± 0.01 g/100 mg), calcium, manganese, and sodium (588.93 ± 7.77, 20.30 ± 0.95, and 57.34 ± 0.33 mg/100 g, respectively) were found. Acetone showed better extraction efficiency than acetone, ethanol, and distilled water. Moreover, acetone flaxseed extract showed higher total phenolic content (84.13 ± 7.73 mgGAE/g), flavonoid content (5.11 ± 1.50 mgQE/g), and FRAP (5031 ± 15.92 μMFe^2+/^g) than fennel seed extract. This study showed that, among both herbs, flaxseed extract may have pharmacological potential in preventing illnesses and may be suggested for use in the food industry as a natural antioxidant.

## Graphical abstract

This current study revealed that flaxseed showed higher protein, fat content, potassium, iron, phosphorus, magnesium, zinc, and amino acids. In fennel seeds, high dietary fiber, calcium, manganese, and sodium were found. Moreover, acetone flaxseed extract showed higher total phenolic content, flavonoid content, and ferrous‐reducing antioxidant power values than fennel seed extract. Finally, among all herbs, flaxseed extract may have pharmacological potential in preventing illnesses and may be suggested for use in the food industry as a natural antioxidant.

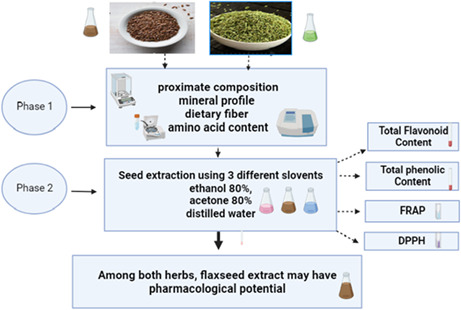

## 1 INTRODUCTION

The recent era has focused on screening medicinal plants/seeds for phytoconstituents, potential agents for usage as medications, and other pharmaceutical items. Tocopherols, carotenoids, ascorbates, polyphenolics, and terpenoids are naturally occurring phytoconstituents from plants that have been investigated and used as alternative therapeutic agents to treat a range of illnesses caused by oxidative stress (Süntar, 2020). Due to their numerous advantageous uses, medicinal plants are highly sought after and prevalent in the functional food and biopharmaceutical sectors (Jaddu & Katam, 2018). Herbs extensively used as medicinal material include cumin, flaxseed, ginger, cardamom, black seeds, and linseeds. Fennel seeds and Flaxseed have anti‐inflammatory qualities and help significantly to treat inflammation.

Fennel (*Foeniculum vulgare*) is a well‐known umbelliferous plant that grows as an annual biennial or perennial. The stem, leaves, and seeds are all edibles. Carbohydrates, moisture, protein, fat, dietary fiber, calcium, phosphorus, iron, salt, potassium, and vitamins are all abundant in fennel seeds (Bukhari et al., 2014). Rutin, chlorogenic acid, eriocitrin, miquelianin, 1,3‐o‐dicaffeoylquinic acid, 1,5‐o‐dicaffeoylquinic acid, 4‐o‐caffeoylquinic acid, 1,4‐o‐dicaffeoylquinic acid, 3‐o‐caffeoylquinic acid, and rosmarinic acid are some of the major phenolic chemicals found in fennel (Križman et al., 2007). In terms of industrial usage, they play a part in the pharmaceutical, cosmetic, fragrance, and food sectors (Venskutonis & Jonusaite, 2014). Flaxseed (*Linum usitatissimum*) belongs to the Linaceae family of plants. It is an annual or biennial plant commercially grown in over 30 countries worldwide (Yang et al., 2021). Flaxseed has long been known for its health advantages due to rich in α‐linolenic acid, dietary fiber, proteins, lignan, omega‐3 fatty acids, flavonoids, phytochemicals, vitamins, and amino acids (Ahmad et al., 2020). Flaxseed contains 41% fat, 20% protein, 28% fiber, 4%–8% moisture, 4% ash, α‐linolenic acid, dietary fiber, proteins, and lignin (Seralthan & Baskaran, 2020). Flax lignans (especially secoisolariciresinol diglucoside, SDG) are now gaining popularity in functional foods and nutraceuticals for their estrogenic and antioxidant properties (de la Bastida et al., 2021).

However, understanding the significance of various medicinal plants in human and animal nutrition and industry requires comprehension of their proximate, minerals, and phytochemical content, as well as the overall mechanism of action of these medicinal herbs. The study aims to quantify the proximate minerals, phytochemical compositions, and antioxidant determination of flaxseed and fennel seeds often found in our region while considering the abovementioned factors.

## 2 MATERIALS AND METHODS

### 2.1 Procurement of raw material

Flaxseed and fennel seeds were purchased from the local market in Lahore, Pakistan. Chemicals and reagents were purchased from Sigma‐Aldrich Tokyo, Japan, Merck (Merck KGaA), and Sigma‐Aldrich. The research was conducted at the University Institute of Diet and Nutritional Sciences (UIDNS), The University of Lahore, Pakistan.

### 2.2 Preparation of seed extracts

The seeds were extracted using three different solvents, i.e., ethanol (80%), acetone (80%), and distilled water. A quantity of 100‐g herbs powder (flaxseed and fennel seeds) was soaked in 2 L of ethanol, acetone, and distilled water for 7 h, followed by centrifugation at 7000 rpm for 15 min. The solvents were recovered using a rotatory evaporator at 400°C after the extracts were filtered using a vacuum filtering assembly (Ahmad et al., 2012).

### 2.3 Chemical composition

Moisture, crude protein, crude fat, crude fiber, crude ash, and nitrogen‐free extract (NFE) of three different herbs (flaxseed and fennel seed powder) were determined according to Association of Official Analytical Chemists and Horwitz (1975).

### 2.4 Dietary fiber content

The enzymatic test was used to assess the total, soluble, and insoluble dietary fiber components in flaxseed and fennel seeds, as described by Sonia et al. (2020).

### 2.5 Mineral profile

Determination of iron, zinc, calcium, magnesium, and manganese was done by atomic absorption spectrophotometer (AAS) (Unicam Model 929), while sodium and potassium were done by flame Photometer (Cole‐Parmer, EW‐83055‐02). Phosphorus was determined by spectrophotometry as described by Gul and Safdar (2009).

#### 2.5.1 Wet digestion of the sample

Wet digestion was carried out in a glass digestion tube, in which 1‐g sample was mixed with 12 ml of HNO_3_ and kept overnight at room temperature. The next day, 4‐ml perchloric acids (HClO4) was added to the mixture and subjected to digestion carried out in a fume block. The block's temperature increased progressively, initially 50°C and increasing progressively to between 250 and 300°C; the temperature was raised. Complete digestion was confirmed by forming white gas within 70–85 min. The mixture was cooled and then added to a 100‐ml volumetric flask by adjusting 100 ml with distilled water, transferred to plastic bottles, and labeled correctly for further mineral analysis (Gul & Safdar, 2009).

#### 2.5.2 Determination of minerals by AAS

Atomic absorption spectrophotometry (Unicam Model 929) was utilized for the evaluation of the mineral composition of digested samples by using different electrodes for each mineral component, including zinc (Zn), iron (Fe), calcium (Ca), magnesium (Mg), and manganese (Mn). Standard solutions of each mineral assessed proper equipment operation before and after the experiment. Except for P and Mg, all minerals had a dilution factor of 100. The original solution was diluted using 0.5 ml of the original solution and 100 ml of distilled water to obtain the Mg content to separate Ca from Mg, and 1 ml of lithium oxide solution was added to the initial solution (Gul & Safdar, 2009). Mineral concentrations measured in parts per million (ppm) were multiplied by the dilution factor, divided by 1000, and then converted to milligrams (mg) as follows:

$$\mathrm{MW}=\frac{\text{Absorbency}\;\left(\mathrm{ppm}\right)\times \mathrm{dry}\;\mathrm{wt}.\times D}{\mathrm{Wt}.\;\text{of sample}\times 1000}$$

#### 2.5.3 Determination of sodium (Na) and potassium (K) by flame photometer

Utilizing flame photometry, the sample was examined for sodium (Na) and potassium (K). The wet‐digested meal sample solutions were used to calculate the salt and potassium levels. Na and K were dissolved in standard solutions of 20, 40, 60, 80, and 100 milli equivalent/L. Estimates for total mineral intake follow AAS's methodology (Gul & Safdar, 2009).

#### 2.5.4 Evaluation of phosphorus (P) by spectrophotometry

A quantity of 250 ml of distilled water and 12 g of ammonium molybdate were combined in a volumetric flask (solution A). In another volumetric flask, 0.2908 g of potassium tartrate of antimony was dissolved in 500‐ml H_2_SO_4_ (5N) solution. One‐litter solution was prepared by adding just enough distilled water (solution B). Two solutions (A and B) were mixed in a 2‐L volumetric flask to create mixed reagents by adding distilled water. A quantity of 140 ml of the combination reagent and 0.739 g of ascorbic acid were put in a beaker and allowed to dissolve to make the color reagent. A wet‐digested duplicate food sample (1 ml) was added to an appropriately labeled plastic container to prepare the diluted sample by adding distilled up to 5 ml. The final solution was prepared by adding 5 ml of color reagent, bringing the total volume up to 25 ml. The dilution factor for this solution was 2500 (100 × 25). The final solution's color eventually turned blue.

Phosphorus analysis: blue colored phosphorus solution was collected in a cuvette for spectrophotometric analysis. Phosphorus levels were measured in ppm, and total mineral intake was also estimated using the same technique described by Gul and Safdar (2009). Mineral concentrations measured in ppm were multiplied by the dilution factor, divided by 1000, and then converted to milligrams (mg) as follows:

$$\mathrm{MW}=\frac{\text{Absorbency}\;\left(\mathrm{ppm}\right)\times \mathrm{dry}\;\mathrm{wt}.\times D}{\mathrm{Wt}.\;\text{of sample}\times 1000}$$

### 2.6 Amino acid profile

To check amino acid determination, ion‐exchange chromatography (IEC) with a Technicon Sequential Multi‐sample (TSM) Amino Acid Analyzer was used, as mentioned by Association of Official Analytical Chemists and Horwitz (1975). Using Soxhlet extraction techniques, a 2‐g sample was defatted by petroleum ether. After that, the consequent sample was dried and ground into fine powder. A sample in duplicate was taken, and 30 mg was weighed into glass ampoules. Then, 5 cm^3^ of 6 M HCl and 5 μmoles norleucine were added into the glass ampoules.

Nitrogen gas was passed through the ampoules to evacuate it. Bunsen burner flame was used to seal the ampoules, and sealed ampoules were hydrolyzed in an oven at 110°C for 24 h. Then, ampoules were left to cool. Once cooled, the tip of the ampoule was broken, and the contents were filtered. Filtered content was dried by evaporating at 40°C in a rotary evaporator. The remaining content was dissolved into 5 μl (for acid and neutral amino acids) or 10 μl (for essential amino acids) with acetate buffer, pH 2.2, and the solutions were distributed into the cartridge of TSM. The chromatograms (amino acid peaks) obtained from the automatic pen recorder relate to the quantity of each amino acid present. The peak areas of each amino acid in the sample were compared to the areas of the corresponding amino acid standards in the protein hydrolysate.

### 2.7 Phytochemical screening assays

Powdered extracts of flaxseed and fennel seeds were dissolved in their respective solvents (ethanol 80%, acetone 80%, and water), and the resulting solutions were used for in vitro analysis, such as phytochemical screening and antioxidant tests.

#### 2.7.1 Determination of total phenolic content (TPC)

Total phenolic content was evaluated using the Folin–Ciocalteu reagent (Manzoor, Zeng, et al., 2019). A Ultraviolet–Vis spectrophotometer was used to produce a calibration curve with a standard solution of gallic acid in the range of 0.01–0.05 μg/ml and measured at 760 nm. All of the tests were done in triplets. The calibration standard was gallic acid, and the findings were stated in milligrams of equivalent gallic acid dry extract (mg GAE/g).

#### 2.7.2 Determination of total flavonoid content (TFC)

Total flavonoid content was determined using quercetin as a standard in phytochemical analysis (Manzoor et al., 2020). To quantify the absorbance, a spectrophotometer was used at 415 nm. The findings were shown as quercetin equivalents in milligrams to the gram of extract (mg QE/g extract). Quercetin in various concentrations (5–50 mg/L) was used to create the standard curve. All of the experiments were run in triplicate.

### 2.8 Antioxidant activity assays

#### 2.8.1 DPPH radical scavenging activity

DPPH is a darkish crystalline powder made of fixed free‐radical molecules. The ascorbic acid concentration was standard, while different extracts were prepared in triplicates to investigate the antioxidant activity. Methanol was used to prepare the control solution instead of the extract. This test measured compounds' and different extracts' hydrogen or electron atomization ability with an illuminating DPPH violet solution. In this method, we made 3.9 ml of DPPH Stokes, added 100 μl of concentrations for each sample, and put it in darkness for 30 min. Absorbance was checked at 516 nm at a Ultraviolet–Vis spectrophotometer. Results were then expressed as IC_50_. The factor IC_50_ is used for the results definition from the DPPH assay and is interpreted as the concentration of substrate that causes a 50% loss of the DPPH activity (Hamedi et al., 2022). The sample's antioxidant activity was determined using the formula below:

$$\text{Antioxidant effect}\%=\left[1-\left(\frac{\text{Sample absorbance}}{\text{Control absorbance}}\right)\right]\times 100$$

Ascorbic acid was used as a standard.

#### 2.8.2 Ferrous‐reducing antioxidant power assay (FRAP)

Following the technique described by Sohail et al. (2018), the reducing power of flaxseed and fennel seed powder was assessed using the ferrous‐reducing antioxidant power (FRAP) test. “One‐milliliter extract was mixed with 1 ml of 1% potassium ferricyanide and 1 ml of 200 mM sodium phosphate, having buffer (pH 6.6). After that, this mixture was incubated at 50°C for 20 min. One milliliter of Trichloroacetic acid (TCA) (10%) was added to this mixture, followed by centrifugation (Hettich, model# 0008017‐10) at 13,400 *g* for 5 min. Furthermore, 1‐ml supernatant was mixed with 1‐ml distilled water and 0.1‐ml ferric chloride (0.1%). The absorbance of the mixture was measured at 700 nm. Aqueous solutions of FeSO_4_.7H_2_O (100–1000 μM) were used for calibration”; values were expressed as micromoles Fe(II) per gram.

### 2.9 Statistical analysis

All assays were done in triplicates, and results are expressed as the mean ± standard deviation. Statistical differences were analyzed by one‐way analysis of variance (ANOVA) at the significance level of *p* ≤ .05 using the program SPSS (25.0).

## 3 RESULTS AND DISCUSSION

### 3.1 Chemical composition

Table 1 depicts the mean values of moisture, ash, crude fat, crude fiber, crude protein, and nitrogen‐free extracts of flaxseed and fennel seeds. The results indicated that higher crude fat and protein were present in flaxseed, whereas fennel seeds constituted higher ash and moisture content. When comparing the outcomes of this present study with the literature; the moisture, ash, protein, fat, and fiber content of flaxseed in the current study were very close to the results presented by Chaudhary et al. (2018), which showed moisture (4.22 ± 0.38%), protein (16.80 ± 0.24%), fat (37.4 ± 0.50%), ash (2.85 ± 0.10%), and fiber (8.27 ± 0.26%) in flaxseed. Additionally, Prajapati et al. (2016) also showed similar results; moisture (5.37%), ash content (3.36%), and fiber (1.22%) in the flaxseed, but another study showed low protein content in flaxseed as compared to another study, which found 28.86% protein content in flaxseed (Amin & Thakur, 2014). Likewise, the crude fat content (36.76%) of flaxseeds is similar to the results from Hanaa et al. (2017).

**TABLE 1:** Chemical composition of flaxseed and fennel seeds (%)

Chemical composition	Moisture	Ash	Fat	Fiber	Protein	NFE
Flaxseed	5.42 ± 0.01^b^	3.67 ± 0.02^b^	36.76 ± 0.02^a^	10.77 ± 0.03^b^	17.33 ± 0.02^a^	26.05 ± 0.21^b^
Fennel seeds	7.83 ± 0.02^a^	11.39 ± 0.02^a^	10.31 ± 0.02^b^	19.14 ± 0.01^a^	11.86 ± 0.01^b^	39.47 ± 0.37^a^

*Note*: Data are represented as Mean ± SD (*n* = 3). Means with different superscript letters are significantly different at *p* ≤ .05.

The result of the current study also showed moisture (7.83 ± 0.02%), protein (11.86 ± 0.01%), fat (10.31 ± 0.02%), fiber (19.14 ± 0.01%), and ash (11.39 ± 0.02%) of fennel seed, respectively. Similar results were found by Saber and Eshra (2019). According to Table 2, total dietary fiber, soluble, and insoluble dietary fiber were higher in fennel seeds, followed by flaxseed; identical results were presented by Venskutonis and Jonusaite (2014), who found total dietary fiber (26.02 ± 031 g/100 mg), soluble (7.81 ± 0.04 g/100 mg), and insoluble dietary fiber (18.20 ± 0.33 g/100 mg), respectively, in flaxseed. Moreover, on the contrary, Bukhari et al. (2014) showed lower total dietary fiber (5.7 g/100 kg) in fennel seeds as compared to the present study.

**TABLE 2:** Dietary fiber of flaxseed and fennel seeds (g/100 g)

Herbs	Soluble dietary fiber	Insoluble dietary fiber	Total dietary fiber
Flaxseed	7.74 ± 0.02^b^	18.06 ± 0.03^b^	25.8 ± 0.32^b^
Fennel seeds	15.96 ± 0.02^a^	37.24 ± 0.02^a^	53.2 ± 0.01^a^

*Note*: Values are represented as Mean ± SD (*n* = 3). Means with different superscript letters are significantly different at *p* ≤ .05.

### 3.2 Mineral determination of flaxseed and fennel seeds

Table 3 shows the mean values of calcium, potassium, sodium, iron, phosphorus, magnesium, zinc, and manganese of flaxseed and fennel seeds. The result of this present study indicated that potassium (763.66 ± 4.04 mg/100 g), iron (5.13 ± 0.03 mg/100 g), phosphorus (581.46 ± 4.07 mg/100 g), magnesium (406.60 ± 5.12 mg/100 g), and zinc (3.30 ± 0.49 mg/100 g) were higher in flaxseed, but calcium (588.93 ± 7.77 mg/100 g), sodium (57.34 ± 0.33 mg/100 g), and manganese (20.30 ± 0.95 mg/100 g) were higher in fennel seeds.

**TABLE 3:** Mineral determination of flaxseed and fennel seeds (mg/100 g)

Minerals	Flaxseed	Fennel seeds
Calcium	230.88 ± 3.05^b^	588.93 ± 7.77^a^
Potassium	763.66 ± 4.04^a^	409.64 ± 3.21^b^
Sodium	16.44 ± 0.77^b^	57.34 ± 0.33^a^
Iron	5.13 ± 0.03^a^	0.94 ± 0.02^b^
Phosphorus	581.46 ± 4.07^a^	47.84 ± 1.50^b^
Magnesium	406.60 ± 5.12^a^	19.44 ± 1.39^b^
Zinc	3.30 ± 0.49^a^	0.41 ± 0.12^b^
Manganese	2.30 ± 0.13^b^	20.30 ± 0.95^a^

*Note*: Data are represented as Mean ± SD (*n* = 3). Means with different superscript letters are significantly different at *p* ≤ .05.

This present study has the same results as Sharma and Saini (2022), who found these mineral content in flaxseed: calcium (220 ± 0.03 mg/100 g), potassium (765 ± 0.21 mg/100 g), sodium (17.00 ± 0.15 mg/100 g), iron (22 ± 0.32 mg/100 g), zinc (2.7 ± 0.93 mg/100 g), and manganese (4.1 ± 1.48 mg/100 g) but magnesium content (765 ± 0.52 mg/100 g) was higher as compared to the current study. Another study showed lower results of calcium (170 ± 0.12 mg/100 g) and phosphorus (370 ± 2.12 mg/100 g) in flaxseed as compared to the present study (Kajla et al., 2015). For fennel seeds, Badgujar et al. (2014) presented the same results of calcium (449 ± 1.22 mg/100 g), potassium (414 ± 2.01 mg/100 g), sodium (52 ± 1.03 mg/100 g), iron (0.73 ± 0.08 mg/100 g), magnesium (17 ± 0.02 mg/100 g), zinc (0.2 ± 0.01 mg/100 g), and phosphorus (50 ± 1.23 mg/100 g). However, Saber and Eshra (2019) revealed higher values of potassium (849.45 ± 3.18 mg/100 g), calcium (583 ± 2.21 mg/100 g), magnesium (85.87 ± 0.94 mg/100 g), iron (9.96 ± 0.61 mg/100 g), and phosphorus (470 ± 2.12 mg/100 g) in fennel seeds, respectively.

### 3.3 Amino acids in flaxseed and fennel seed extracts

Table 4 presents amino acids in g/100 g, alanine (6.49 ± 0.15), arginine (13.92 ± 0.00), glycine (2.11 ± 0.00), histidine (6.90 ± 0.00), isoleucine (6.28 ± 0.00), leucine (4.86 ± 0.57), lysine (2.78 ± 0.00), methionine (2.80 ± 0.00), phenylalanine (4.15 ± 0.00), proline (3.43 ± 0.57), serine (6.70 ± 0.00), threonine (6.86 ± 0.05), tyrosine (5.10 ± 0.00), valine (3.43 ± 0.05), glutamic acid (8.45 ± 0.11), and aspartic acid (6.51 ± 0.01) were high in flaxseed followed by fennel seeds. At the same time, cysteine (0.23 ± 0.00) and tryptophan (0.57 ± 0.01) were high in fennel seeds, followed by flaxseed. de la Bastida et al. (2021) showed similar results regarding the amino acid content of flaxseeds.

**TABLE 4:** Amino acids in flaxseed and fennel seeds (g/100 g)

Amino acids	Flaxseed	Fennel seeds
Alanine	6.49 ± 0.15^a^	0.83 ± 0.06^b^
Arginine	13.92 ± 0.00^a^	0.630 ± 6.80^b^
Cysteine	0.11 ± 0.01^b^	0.23 ± 0.00^a^
Glutamic acid	8.45 ± 0.11^a^	2.91 ± 0.00^b^
Glycine	2.11 ± 0.00^a^	0.51 ± 0.00^b^
Histidine	6.90 ± 0.00^a^	0.13 ± 0.00^b^
Isoleucine	6.28 ± 0.00^a^	0.73 ± 0.57^b^
Leucine	4.86 ± 0.57^a^	0.61 ± 0.00^b^
Lysine	2.78 ± 0.00^a^	0.74 ± 0.11^b^
Methionine	2.80 ± 0.00^a^	0.20 ± 0.17^b^
Phenylalanine	4.15 ± 0.00^a^	0.42 ± 0.00^b^
Proline	3.43 ± 0.57^a^	0.14 ± 0.00^b^
Serine	6.70 ± 0.00^a^	0.92 ± 0.00^b^
Threonine	6.86 ± 0.05^a^	0.69 ± 0.00^b^
Tryptophan	0.12 ± 0.00^b^	0.57 ± 0.01^a^
Tyrosine	5.10 ± 0.00^a^	0.43 ± 0.00^b^
Valine	3.43 ± 0.05^a^	0.95 ± 0.00^b^
aspartic acid	6.51 ± 0.01^a^	1.88 ± 0.01^b^

*Note*: Data are represented as Mean ± SD (*n* = 3). Means with different superscript letters are significantly different at *p* ≤ .05.

### 3.4 Quantitative analysis of phytochemicals

**FIGURE 1:**
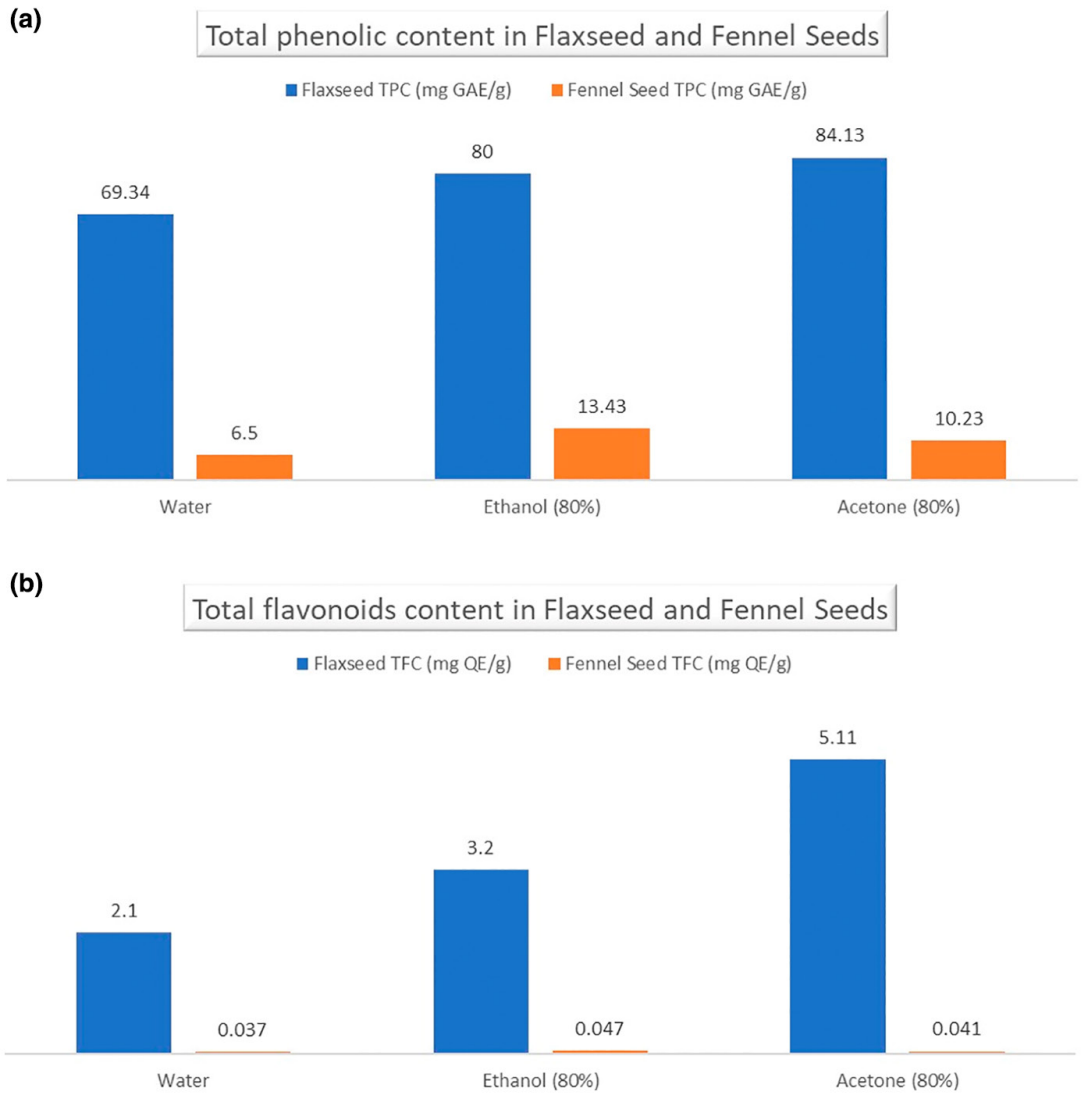
(a) Total phenolic in flaxseed and fennel seeds, (b) flavonoids content in flaxseed and fennel seeds.

Phytochemicals have excellent antioxidant potential and beneficial impacts on human health (Manzoor, Ahmad, et al., 2019; Manzoor et al., 2021; Manzoor, Hussain, Naumovski, et al., 2022). Figure 1a depicts the TPC of flaxseed and fennel seed extracts with three solvents (distilled water, 80% ethanol, and 80% acetone) presented as gallic acid equivalents (GAE). Flaxseed water extract showed lower total phenolic acid content (69.34 ± 1.28 mgGAE/g), whereas acetone flaxseed extract showed the highest phenolic acid content (84.13 ± 7.73 mgGAE/g). The same results were found by observing the TPC value of water and acetone at 71.7 and 78.4 mg GAE/g (Barthet et al., 2014). Fennel water extract also showed lower total phenolic acid content (6.5 ± 0.81 mgGAE/g), while ethanolic extract (13.43 ± 3.58 mgGAE/g) showed the highest phenolic acid content. The same results were confirmed by Rajić et al. (2018), who found the ethanolic extract of fennel seed to be higher, and Beyazen et al. (2017) mentioned that water extract has a low TPC value as compared to methanol.

**FIGURE 2:**
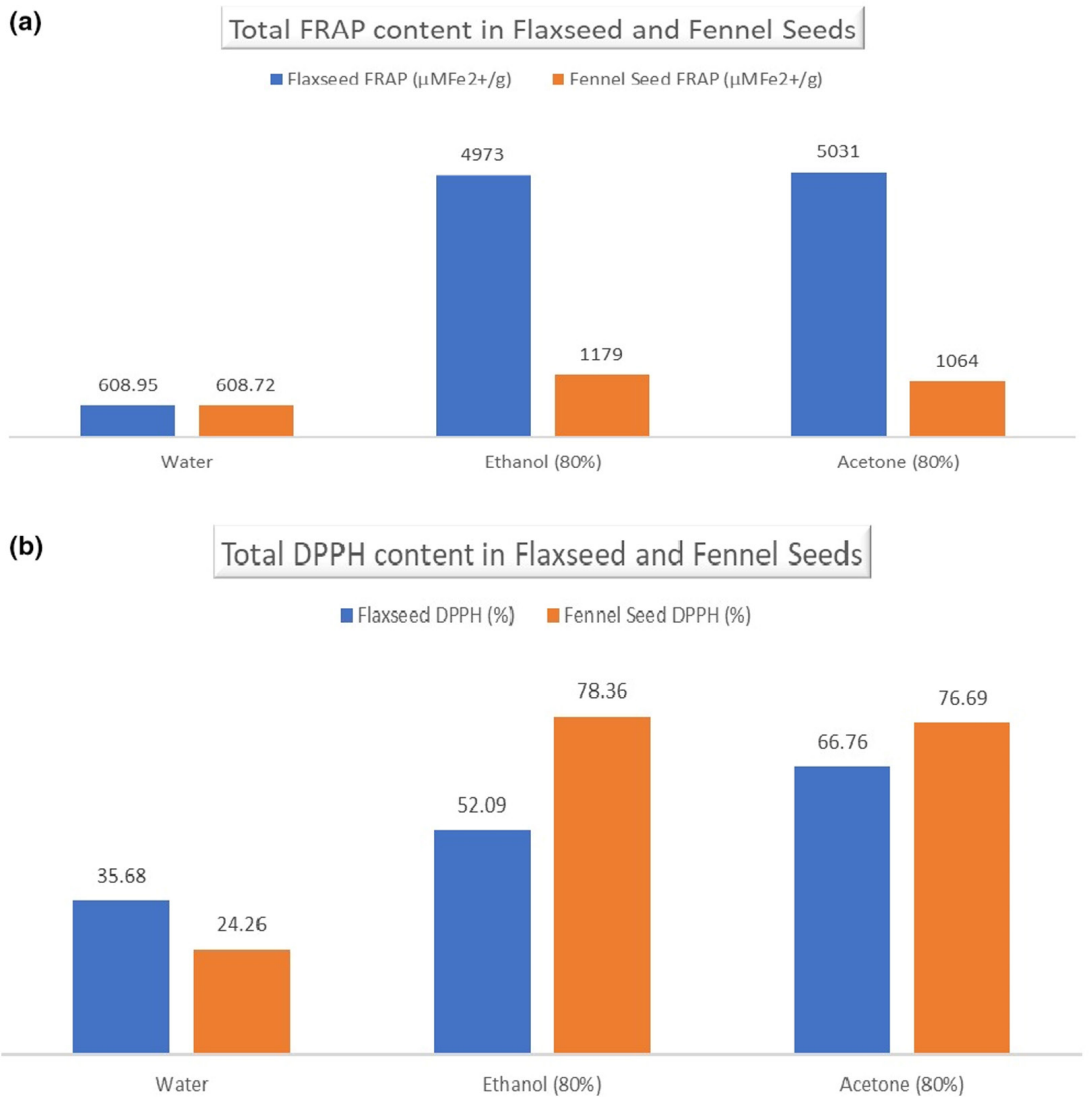
(a) Total FRAP contents of flaxseed and fennel seeds, (b) total DPPH contents of flaxseed and fennel seeds.

Figure 2b portrays the total flavonoid content of flaxseed and fennel seed extract with three solvents (distilled water, 80% ethanol, and 80% acetone) expressed as quercetin equivalent (QE). Flaxseed water extract showed lower total flavonoid content (2.1 ± 0.59 mg QE/g), while flaxseed acetone extract (5.11 ± 1.50 mg QE/g) showed the highest flavonoid content. Identical results were found by Hanaa et al. (2017), who presented that total flavonoid contents were high in acetone and less in water, and alternate results were found by Punia and Deen (2016), who mentioned that the TFC of water extract flaxseed was 92.00 ± 2.34 mg/g and flaxseed ethanol extract was 185.00 ± 1.04 mg/g. Water extract depicted lower total flavonoid content, while ethanolic extract showed the highest flavonoid content, and the same results were found by Goswami and Chatterjee (2014).

### 3.5 Antioxidant activity of flaxseed and fennel seeds

Plants are a good source of antioxidants, and their antioxidant properties are associated with hydroxyl groups in their structural formulae (Ali et al., 2022; Manzoor et al., 2017; Manzoor, Hussain, Tazeddinova, et al., 2022). In the present study, the antioxidant activity of ethanol 80%, acetone 80%, and water extracts of flaxseed and fennel seeds were estimated by the radical scavenging activity (DPPH), and ferric‐reducing power (FRAP) was analyzed with a maximum absorption band of around 515–517 nm (Figure 2). It is frequently used to assess the antioxidant capacity of compounds (Al‐Radadi, 2021). The study's findings revealed a considerable difference between the extracts. The DPPH range of flaxseed was 35.68%–66.76%. Radical scavenging activity toward DPPH was the highest for acetone extract, followed by ethanol and water extract. Similar results were found by Barthet et al. (2014). In comparison, alternate results were found by Waszkowiak et al. (2015), who presented that DPPH with water extract was higher than ethanolic flaxseed extract. At the same time, the DPPH range of fennel seeds was 70.26%–95.69%. Radical scavenging activity toward DPPH was the highest for ethanol extract, followed by acetone and water extract. Analogous results were found by Goswami and Chatterjee (2014), who mentioned that ethanol extract has a higher value of DPPH as compared to water extract (96.2 and 84.61), and Khammassi et al. (2022) also presented that fennel water extract has a low DPPH value as compared to 80% ethanolic extract (21.68% and 71.24%).

The FRAP assay determined the antioxidant activity of flaxseed and fennel seed extract, a stable organic free radical with a maximum absorption band of around 700 nm (Khammassi et al., 2022). The antioxidant activity of flaxseed extracts ranged from 608.9 to 5031 μM Fe2+/g. Flaxseed acetone extract presented the highest FRAP value compared to water flaxseed extract. The same results were found by Waszkowiak et al. (2015). The antioxidant activity of fennel seed extract ranged from 608.72 to 1197 μM Fe2+/g. Methanolic extract of fennel seed depicted the highest FRAP value compared to acetone and water flaxseed extract; a comparable result was found by Goswami and Chatterjee (2014).

## 4 CONCLUSION

Due to the adverse effects of manufactured pharmaceuticals, awareness of using native plants as traditional medicine for diseases has grown. Even though fennel seeds and flaxseed have been used as herbal medicine for many years, little is known about their nutritional and phytochemical content. The current study revealed that fennel seeds and flaxseed were good sources of essential phytochemicals and phytonutrients, consistent with their ethno‐medicine usage. Flaxseed might be essential to your daily diet because of its unique nutritional value and antioxidant activity. As a result, using this seed as a functional, health‐enhancing meal should be advocated, notably for preventing diabetes, obesity, hyperlipidemia, cancer, and neurogenerative diseases which affect millions of people worldwide.

## CONFLICT OF INTEREST

The authors declare that they have no conflict of interest.

## Data Availability

The dataset supporting the conclusions of this article is included within the article.
